# Non-degree allopathic practitioners as first contact points for acute illness episodes: insights from a qualitative study in rural northern India

**DOI:** 10.1186/1472-6963-14-182

**Published:** 2014-04-23

**Authors:** Christina May, Katja Roth, Pradeep Panda

**Affiliations:** 1 Department for Cooperative Studies, University of Cologne, Albertus-Magnus-Platz, 50923 Cologne, Germany; 2 Micro Insurance Academy, 52-B, Okhla Industrial Estate, Phase III, 110020 New Delhi, India

**Keywords:** Rural health care, Access to health care, Informal health providers, Non-degree allopathic practitioners, India

## Abstract

**Background:**

In 2005, the Indian government launched the National Rural Health Mission (NRHM) to improve the quality of and access to rural public health care. Despite these efforts, recent evidence shows that the rural poor continue to primarily consult private non-degree allopathic practitioners (NDAPs) for acute illness episodes. To examine this phenomenon, we explore the rural poor’s perception and utilization of the rural health care system and the role and accessibility of NDAPs therein.

**Methods:**

Our study is based on qualitative data from focus group discussions conducted in three rural districts in Bihar and Uttar Pradesh, two high-focus states of the NRHM in northern India, in 2009/2010. Our study population consists of female micro-credit self-help group members and their male household heads. We apply a directed content analysis and use a theoretical framework to differentiate between physical, financial and cultural access to care.

**Results:**

Our study population distinguishes between “home treatment” (informal self-care), “local treatment” (formally unqualified care) and “outside treatment” (formally qualified care). Because of their proximity, flexible payment options and familiarity with patients’ belief systems, among other things, local NDAPs are physically, financially and culturally accessible. They are usually the first contact points for patients before turning to qualified practitioners, and treat minor illnesses, provide first relief, refer patients to other providers and administer formally prescribed treatments.

**Conclusion:**

Our findings are similar for all three study sites and reinforce recent findings from southern and eastern India. The poor’s understanding and utilization of the rural health system deviates from governmental ideas. Because of their embeddedness in the community, private NDAPs are the most accessible medical providers and first contact points for acute illness episodes. Thus, they de-facto fulfill the role envisaged by the Indian government for accredited social health activists introduced as part of the NRHM. We conclude that instead of trying to replace NDAPs with public initiatives, the Indian government should regulate, qualify and integrate them as part of the existing public health care system. This way, we argue, India can improve the rural poor’s access to formally qualified practitioners.

## Background

India’s National Rural Health Mission (NRHM), launched in 2005, aimed to improve the quality of and access to public health care services, especially for the rural poor. The reasons for these measures were the manifold problems in the public sector hampering the utilization of its services, such as low-quality care, absenteeism of staff and a dearth of physically accessible facilities [1]. The results of the NRHM have been mixed: availability of health resources and health services delivery have improved [1,2], but problems with the quality of care continue [3]. After its first 7 years, the initiative failed to reach all of its key goals and was hence extended until 2017 [4,5].

Despite the achievements of the NRHM with regard to availability of public health resources, recent evidence shows that the rural poor seek care for acute illness episodes primarily from private non-degree allopathic practitioners (NDAPs) [6,7]. These health care providers practice allopathy, although they do not have valid qualification in modern medicine. Some do not have any kind of qualification, while others hold degrees from traditional Indian systems of medicine (Ayurveda, Yoga and Naturopathy, Unani, Siddha and Homeopathy, often summarized under AYUSH) [8-13]^a^. Our article addresses the question why the rural poor continue to rely on NDAPs. We explore the NDAPs’ accessibility and characteristics from the viewpoint of their patients and examine their role in the rural health care system. To better comprehend this role, we consider the communities’ understanding of the local health system and the different health care options they perceive. Recent studies on the utilization of rural health care after the introduction of the NRHM use a quantitative approach [7], focus on southern and eastern India [6,14] or do not differentiate between formal and informal health care providers [15]. We use data from a qualitative study conducted in three rural settings in Bihar and Uttar Pradesh, two of the high-focus states of the NRHM in northern India. We focus on acute illness episodes because they are the most frequent ones. Also, as a household survey of our study population has shown, NDAPs are the most popular health care providers for these illnesses [7]. To explain NDAPs’ significance, we use a theoretical framework for access to health care based on Penchansky and Thomas [16] and adapted by McIntyre et al. [17] and Peters et al. [18].

In the next sections, we give an overview of the rural health care system in India and present the theoretical framework that guides our analysis. This is followed by a description of the data and methods used before we present and discuss our results. The article ends with some concluding remarks.

### Rural Indian health care system

Rural Indian health care in the public sector is hierarchically organized in a three-tier system and based on population norms within a geographical area (see Table 1). Higher-level facilities serve as referral units for lower-level facilities. Sub-centers staffed with one male and at least one female health worker represent the first level and are the first contact point for primary care. They engage in promoting behavioral change that is integral to maternal and child health, family welfare, nutrition, immunization, and prevention of diarrhea and communicable diseases. Sub-centers are equipped with basic medicines for minor ailments [19]. As part of the NRHM, the Indian government introduced so-called Accredited Social Health Activists (ASHAs) – female community health workers – as a contact point in the villages for any health-related demand, but especially for those of women and children. ASHAs are designed to act as a link between the community and the public health system, promoting good health practices, providing basic curative services and making referrals [20]. Primary Health Centers (PHCs) represent the second level of the rural public health care system. They are the first point of contact with a qualified doctor. Here, the medical officer and other paramedical staff provide curative, preventive, promotive and family welfare services. Community Health Centers (CHCs) are the third level. They serve as referral centers for PHCs and offer specialist services and testing facilities [19]. Fees are charged in public hospitals and health centers, but people living below poverty line are exempted [21,22], though the High Level Expert Group on Universal Health Coverage in India expressed its doubt about the practical implementation of these exemptions [23].

**Table 1:** Population norms for public health facilities in rural areas in India

	**Norm in plain areas**	**Norm in hilly/tribal/difficult areas**
Sub-Center	5 000	3 000
Primary Health Center	30 000	20 000
Community Health Center	120 000	80 000

(Source: adopted from MoHFW, Rural health statistics in India, 2012, p.3).

As of March 2010, at the time of our data collection, only 7 out of 35 Indian states and territories fulfilled the population norms for sub-centers, PHCs and CHCs as defined by the Ministry of Health and Family Welfare. Bihar was 35.18% short of required sub-centers, 25.15% short of required PHCs and 88.75% short of required CHCs; Uttar Pradesh was 22.1% short of required sub-centers, 15.9% short of required PHCs and 53.05% short of required CHCs. In those PHCs already functioning, only 20 out of 35 states/territories had no shortfall of allopathic doctors; Uttar Pradesh had a shortfall of 45.77% and Bihar a shortfall of 11.95%. Only two states (Sikkim, Chandigarh) had no shortfall of specialists at already functioning CHCs; Uttar Pradesh had a shortfall of 70% and Bihar a shortfall of 62.86% [24]. According to the latest data from 2012, the availability of doctors has improved in our study states, but is still deficient. The shortfall of allopathic doctors in PHCs is still 22.51% in Uttar Pradesh, while it has decreased to zero in Bihar; the shortfall of specialists at CHCs is 15.53% in Uttar Pradesh and 46.07% in Bihar [19].

Apart from public facilities, a vast number of private health care providers are available in rural India, but many of them have insufficient training. In a national study involving 812 private medical practitioners in 507 Indian villages, only 11.1% of these providers had a formal qualification in allopathy (Bachelor of Medicine or higher). Nevertheless, allopathy is the most dominant form of treatment: 71.6% of all providers surveyed reported that they practiced it [12]. Non-national studies from across India report similar findings with regard to the lack of formal qualification of rural medical practitioners and the prevalence of allopathic treatment [9-11,25-27]. NDAPs administer, prescribe and sell medicine, injections and intra-venous fluids and do minor surgeries [9,25,27,28].

Despite their lack of formal qualification, NDAPs are preferred by the community because they permit deferred payment or payment in kind, are located nearby and available around the clock and offer fast, friendly and effective treatment with powerful medications and injections [8,28]. Recent studies conducted after the introduction of the NRHM confirmed that NDAPs are still perceived by patients as being affordable, accessible and providing quick “all-in-one” services [6,29]. George and Iyer [14] had a closer look at the relationship between NDAPs and their communities in northern Karnataka, India. They found that NDAPs and other informal health providers are embedded in their communities, and stress the social pressure these providers are exposed to – one of the reasons they prescribe allopathic medicine. Banerjee and Duflo also report under - and overmedication by NDAPs as a result of patients’ demand for cheap and quick recovery [30]. Because of their popularity and lack of formal training, demands for new efforts in certifying, integrating and regulating NDAPs have been brought forward since the 1990s [8,12,28,31,32].

### Theoretical framework for analysis

In order to understand the community’s preference for NDAPs, it is important to have a look at their accessibility. In analyzing our data, we applied the theoretical framework described below in this section.

Andersen differentiates between potential access (the presence of resources enabling an individual to use health care services) and realized access (the actual use of health care services) [33]. Realized access or utilization is often used as an indicator for access to health care because it is argued that access without utilization is not an end itself [34,35]. Others have claimed that potential access is the appropriate focus because it can be a value in itself for individuals, even if they do not make use of it [36]. As our focus is on actual utilization of NDAPs, we follow the concept of realized access.

One influential framework of access was developed by Penchansky and Thomas [16]. It consists of five dimensions – availability, accessibility, accommodation, affordability and acceptability – to evaluate the “degree of fit” between the clients and the health system. As some components of this original framework are not always easy to distinguish, we define the following dimensions, a combination of adaptations by McIntyre et al. [17] and Peters et al. [18]^b^:

• Availability or physical access: the supply with appropriate health care providers or services in the right place at the right time

• Affordability or financial access: the fit between costs of service utilization and the patient’s ability to pay

• Acceptability or cultural access: the fit between provider and patient attitudes; responsiveness of health services and providers to social and cultural expectations of their patients and communities

## Methods

### Sampling and data collection

The data presented here is part of a larger study on the impact of three community-based health insurance (CBHI) schemes in the Vaishali district (Mahua block) in Bihar, as well as in the Pratapgarh district (Shivgarh block) and the Kanpur Dehat district (Rasoolabad block), both in Uttar Pradesh, India. Bihar and Uttar Pradesh are two of the high-focus states of the NRHM with weak public health infrastructure and indicators. This larger study is a collaboration between Erasmus University Rotterdam, Netherlands; the University of Cologne, Germany; the Micro Insurance Academy, India; and three local Indian partner organizations (BAIF, Nidan, Shramik Bharti). It combines quantitative, qualitative and spatial data and a stepped wedge implementation of CBHI schemes [37,38]. The data used in this article was collected prior to the introduction of these schemes. The study population consists of members of female micro-credit self-help groups (SHGs) and their households. These SHGs are groups of 10-12 women saving together and giving each other loans from their common fund. They are facilitated by one of the three local partner organizations. On the whole, the SHG households are economically and socially disadvantaged in comparison to non-SHG households from the same villages [39]. At the same time, they are privileged in comparison to non-SHG households when it comes to access to credit for health care: in case larger amounts are needed, our respondents can turn to their respective SHG for a loan at a relatively low interest rate. Using data from a census of all SHG-affiliated households eligible for the CBHI schemes and from a spatial survey, both conducted as parts of the larger study mentioned above, Table 2 compares the socioeconomic characteristics of the SHG households and the average distance to the next public formally qualified doctor in a PHC/CHC. SHG households in the Kanpur Dehat district are on average socioeconomically better off than those in the other two sites, with better educated household heads, a higher average monthly per capita expenditure and a lower proportion of scheduled tribes/scheduled castes households. At the same time, the Kanpur Dehat district is the site with the largest average distance to the next PHC/CHC. Due to these differences, we undertook sampling, data collection and data analysis separately for all three sites to achieve greater external validity.

**Table 2:** Socioeconomic characteristics of study households

	**SC/ST (in percent)**	**Average years of household head’s education**	**Average MPCE (in INR)**	**Average distance of study villages to next PHC/CHC (in km)**
Kanpur Dehat district, Uttar Pradesh	28.6	6.2	1781.1	5
Pratapgarh district, Uttar Pradesh	42.5	5.3	1194.7	3.1
Vaishali district, Bihar	33.8	4.1	1269.8	2.9

SC/ST: Scheduled caste/scheduled tribe – historically disadvantaged groups in India.

MPCE: Monthly per capita expenditure. INR: Indian Rupee.

N = 1,039 SHG households (Kanpur Dehat district), 1,284 SHG households (Pratapgarh district), 1,363 SHG households (Vaishali district).

Data collection took place in December 2009 and January 2010. We conducted focus group discussions (FGDs) with members of the SHGs and their respective household heads. To account for the differences in geographic accessibility of public health care providers among the SHGs, we purposefully chose respondents from villages with close, medium and far distances to the next PHC or CHC in each study site. CHCs were used as reference facilities in the Kanpur Dehat and Pratapgarh districts. In the Vaishali district, there was no CHC in our study block at the time of data collection, so PHCs were used as reference facilities. We did not use sub-centers as reference facilities because these are not staffed with formally qualified doctors. We then worked together with the respective local partner organization to select those SHGs most likely to be willing to participate in the study from all SHGs active in the identified villages. The broad thematic approach of the overarching CBHI study, which comprises various impact-related topics not addressed in this article but covered in the same FGDs, made it necessary to identify a number of respondents large enough to reach data saturation in each region. Otherwise, our data would have been at risk of not being rich enough for a meaningful qualitative analysis [40]. We conducted FGDs separately by gender and within a single self-help group only. In the Kanpur Dehat district, 18 FGDs with female participants and 18 with male participants were held; in the Pratapgarh district, 18 FGDs with female participants and 17 with male participants were held; and in the Vaishali district, 12 with female participants and 17 with male participants were held. Table 3 shows the number of FGDs conducted at each site and for each distance category.

**Table 3:** Number of FGDs conducted, separated by site and distance

	**Kanpur Dehat district**			Pratapgarh district*			Vaishali district*		
**Distance (in km)**^**+**^	**Close**	**Medium**	**Far**	**Close**	**Medium**	**Far**	**Close**	**Medium**	**Far**
	**< 10**	**11-20**	**> 20**	**< 5**	**5-6**	**≥ 7**	**≤ 3**	**4**	**≥ 5**
Female SHG members (FGDs)	6	6	6	7	5	6	4	4	4
Male household heads (FGDs)	6	6	6	4	7	6	5	6	6

^+^For Kanpur Dehat district and Pratapgarh district, the distance reflects the proximity to the next CHC; for Vaishali district, the proximity to the next PHC.

*The numbers of female and male FGDs do not match because some discussions with female/male groups could not be arranged.

To allow for data comparison, we used semi-structured FGD guidelines containing both exploratory questions and targeted questions. Participation in all interventions was voluntary and confidential, based on informed consent. FGDs were conducted by a qualitative researcher from the Micro Insurance Academy and researchers from the local partner organizations, who were trained in a four-day workshop that included recorded mock-sessions. All discussions were conducted in Hindi and tape-recorded; transcripts were then translated into English.

In accordance with the guidelines issued by the Indian Council of Medical Research [41], the overall study and the English versions of all employed data collection tools were checked and approved by the Ethics Committee of the University of Cologne (Germany).

### Data analysis

Using NVivo software, we conducted a directed qualitative content analysis of our data [42]. The analysis was deductive, but also involved inductive categorization of data. Its process consisted of two steps: first, we derived codes and a hierarchical coding tree deductively from the literature and the FGD guidelines. Coding was done by three researchers cross-site, and we added inductive codes or adapted existing ones whenever the content of the FGDs did not fit into one of the established codes. Comparisons between the codings of different researchers ensured a common understanding of text and codes. After this step, we focused on codes relevant to the research question under study in this article. From these, we developed interpretative codes, again comprising deductive codes using literature on health-care-seeking behavior and inductive codes describing patterns and “indigenous typologies”, i.e. classifications developed by the population under study [43]. We conducted the analysis separately for each site to achieve greater external validity, but found a common understanding of the rural health care system and the favorable characteristics of NDAPs. The results from the three sites are therefore presented together; differences found between them are addressed when present in the data.

## Results

### Three levels of health care: home, local and outside

Before turning to non-degree allopathic practitioners and their accessibility in the three dimensions defined in our theoretical framework, we want to understand the broader context of the rural health care system as it is perceived and utilized by our target population. This will then help us in defining the role NDAPs play in the system.

**Figure 1:**
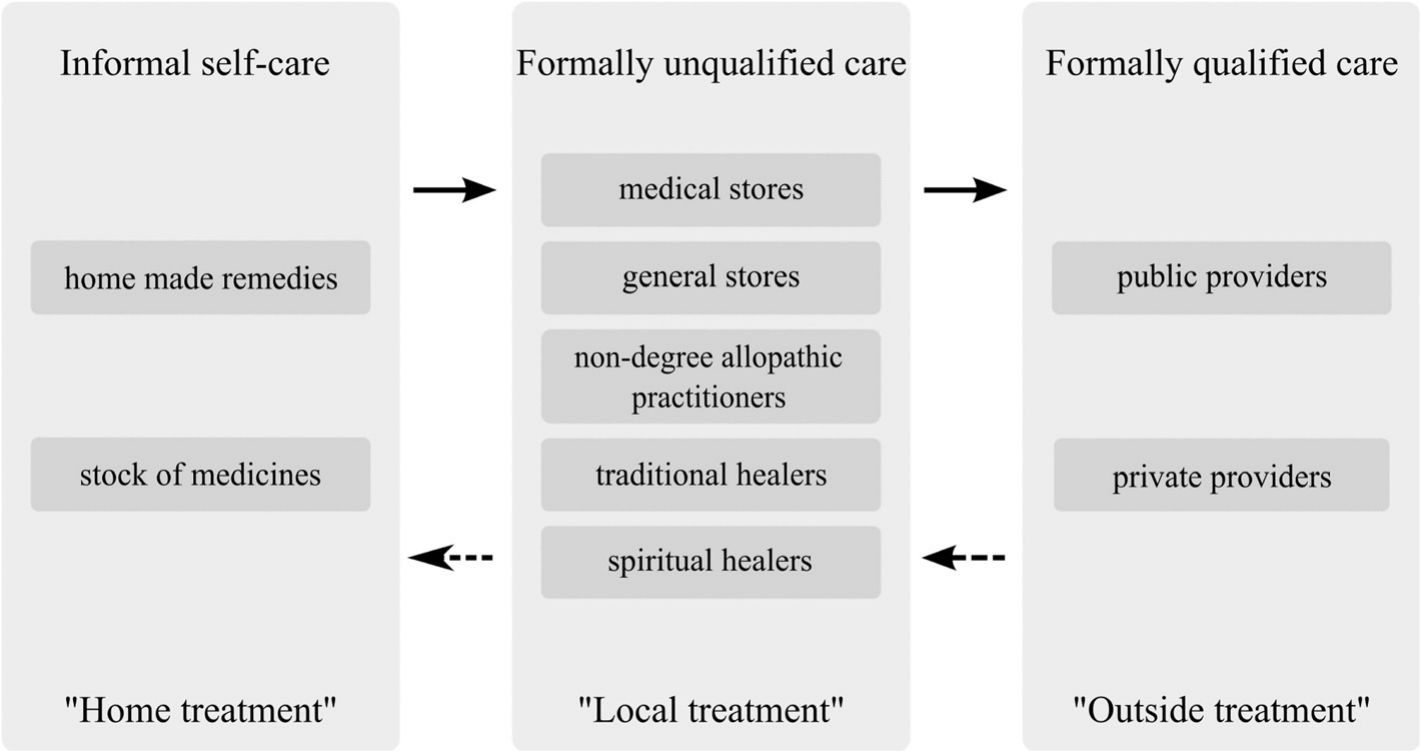
Different health care levels in rural northern India as described by the study population.

The notions used by our respondents to structure and describe different health care sources available reveal an indigenous typology. The central criterion for this typology is location. Respondents group sources of health care into three levels: “home treatment”, “local treatment” and “outside treatment”, characterized by informal self-care, care by formally unqualified practitioners and care by formally qualified practitioners, respectively (see Figure 1).

The first level refers to care provided by family members or the patients themselves at home. This can include home remedies as well as self-medication: “For normal sickness why visit medical stores? We get treated at home” (FGD with female SHG members, Pratapgarh district).

The second level consists of formally unqualified care provided by a variety of practitioners. These are usually available “locally”, “in the village”, “here” or “nearby”: “Here, if someone has fever or something, we call the local doctor” (FGD with female SHG members, Vaishali district). Despite their lack of formal training, respondents usually refer to these practitioners as “doctors”. From the FGDs we learned that these are non-degree allopathic practitioners offering mostly allopathic treatment, though sometimes mixing it with medicines from other systems of medicine.

Specialized traditional and spiritual healers, another source of health care on the local level, are not as popular as they used to be and respondents reported that most villagers turn to allopathic, modern treatment nowadays. Specialized medical stores or general stores selling common medicines are another important source of local health care services. They offer inexpensive and often immediate relief for minor ailments such as coughs, colds, fevers, diarrhea and headaches. Specialized medicines are not available at these shops, though, and need to be procured outside the village. Interestingly, female community health workers, ASHAs, – supposed to be the primary contact point for health-related demands in the villages – were not mentioned as a local health care option for acute illness episodes by our respondents. Mostly, they were mentioned in relation to other services such as maternal care, family planning and public health programs for tuberculosis.

The third level of care is the formal health care system, i.e. formally qualified public and private providers such as doctors, nurses and other paramedical staff. In contrast to the other two levels, these providers are usually not located in the villages and respondents refer to them as being “outside”. One respondent said, “Even in case of some major disease, we call him [the local doctor] and if he can’t control the disease, we have to consult some other doctors outside” (FGD with female SHG members, Vaishali district). At this level, both public and private providers such as clinics, PHCs, CHCs and pharmacies are approached by the patients often only after the illness has progressed and other treatments have not helped: “Firstly we go to local doctors. (…) If it does not get cured in 2 to 3 days, then we take the patient to the hospital” (FGD with female SHG members, Pratapgarh district).

Our study group established a pathway along an informal hierarchical system (illustrated in Figure 1), similar to the hierarchical structure of the public rural health system guiding patients from sub-centers to CHCs. Both systems overlap only partially because patients seek health care in informal and untrained settings including non-allopathic and other healing services. The pathway leads from lower levels to higher levels: from self-care at home to consultation of local formally unqualified practitioners and onwards to formally qualified ones: “If there is less severe pain, then someone within the family advises to take one or two dosages of drugs from the medical store and you will feel relieved or we will see what to do. Men decide to visit the doctor based on [the] amount they have in [their] wallet. They will first go to [the] store, if they do not feel better after taking medicines from the store, then they visit the doctor. If the small-time doctors [NDAPs] are unable to handle the case, then villagers go to [a] nursing home. If the nursing home is also unable to handle the case, then the villagers go to the Pratapgarh hospital [district hospital] or bigger nursing home in Pratapgarh” (FGD with male household heads, Pratapgarh district). There are also many instances when people move laterally, i.e. from one provider to another on the same level, due to dissatisfaction about lack of improvement.

Formally qualified providers, whether public or private, are often the last resort when other options have failed. For minor illnesses, but also for very urgent ailments that cannot be delayed, care is first sought at the local level. But respondents do not always enter the pathway described at the lowest level. For more serious cases such as high fever care is directly sought outside if the means are available, either from public or private formally qualified providers. Respondents say they choose the particular provider based on the distance and costs of the health care sources considered suitable for their ailment. In some cases distance and costs prevent respondents from accessing care at a higher level: “Where will we go [other than the NDAP] when we do not have resources? (…) If some problem comes in the evening, then where will we go? Then we call [the NDAP], he gives some medicine. Once we relax a bit, then we go to Birhun [nearby town] the next morning” (FGD with female SHG members, Kanpur Dehat district).

The predominant reason mentioned for turning to one of the next levels of care is the current caregivers’ inability to cure the ailment. When home treatment fails, households seek relief from informal or formal health care providers. If the condition persists after consulting a NDAP or more advanced services are needed, patients proceed to formally qualified providers, either on their own or through referrals from the local provider. Referrals can be to specific hospitals or doctors (both public and private) or without specific recommendation. Sometimes NDAPs make referrals immediately after realizing that they are unable to treat a condition. Often, however, they attempt to provide treatment and refer patients to other providers only when conditions worsen. Few respondents believe that NDAPs receive commissions for referrals to larger hospitals.

### Comparison of structure and pathway across study sites and distances

Since the proximity of formally qualified doctors differed for the population under study, the classification of providers was not always uniform. A few respondents identified public, formally qualified providers as “local” because they are located very close to their village. Yet the perception that providers from outside are better trained than local providers, including local public doctors, holds true for many respondents: “Whatever these people are telling is right. All the doctors that come over here are not perfect. (…) There are no experienced doctors available here” (FGD with male household heads, Pratapgarh district, close proximity to next CHC). At the same time, respondents also reported visiting NDAPs and other informal providers who are from “outside” their local area, such as famous spiritual healers. They also gave examples of trained health care providers unable to cure a particular condition, despite their formal education and the use of sophisticated and costly medical services, and how local informal providers were able to help. In general, however, the understanding of the health care system as a three-level hierarchical structure described above is strongly supported by the data.

The common pathway described by our respondents was similar for all three study sites and all distances to the next PHC/CHC. Our data suggests that qualified doctors are less accessible geographically in the Kanpur Dehat district than in the other two sites, which is in accordance with the average distances to the next PHC/CHC given in Table 2. As for the use of spiritual healers, respondents from the Kanpur Dehat and Vaishali districts reported a decline in visitations, though a female respondent from the Pratapgarh district mentioned that the number of spiritual healers available in the village had risen.

### Popularity and accessibility of non-degree allopathic practitioners (NDAPs)

Even when respondents live close to formally qualified public doctors, the use of NDAPs is a consistent pattern in our FGDs. This suggests that other factors in addition to distance determine the choice of provider. In the following, we group our respondents’ explanations for the utilization of NDAPs into the three dimensions of accessibility defined before.

#### ***Availability or physical access***

Our study population identifies NDAPs as “jholachap”, a Hindi expression literally translating into “man with a bag” and a reference to their accessibility as they travel around to offer their services. NDAPs are situated close to the population and often available around the clock. This makes them a first contact point for acute illness episodes. People save time when they do not have to travel to far away and often overcrowded public facilities with long waiting periods. Instead, they acquire services conveniently at their doorsteps. NDAPs also make home visits and can be reached by the respondents via mobile phone.

Our findings indicate that the rural population is aware of the lack of formal qualification of NDAPs and the fact that formally qualified modern practitioners are not available at the local level. A female SHG member from the Kanpur Dehat district explained the difference between formally trained health care providers and NDAPs: “The doctor here keeps giving the medicines for pain without knowing the reason of pain. It is only after we go to some doctor to another place that we will know the cause of illness.” Rural residents nevertheless make use of these providers because of their availability. Respondents complained that NDAPs sometimes treat conditions that they know are beyond their capabilities.

Patients also use NDAPs as a first point of relief while they accumulate the funds needed to see a formally qualified health care provider. In these cases, NDAPs are not expected to cure the condition, but are seen as a temporary solution until better options can be accessed: “We have to manage the sources like we call the doctors from [the] village and get some injections and medicines [in case of illness]. After some relief we again proceed to the better option available” (FGD with male household heads, Kanpur Dehat district).

#### ***Affordability or financial access***

NDAPs are more affordable than “outside” private providers. They usually provide their services for a lump-sum, and charge only for medication without taking consultation fees, though some respondents reported that NDAPs charge additional fees for home visits. Our respondents prefer NDAPs to governmental hospitals and primary health centers. Although the latter offer services at a lower price than NDAPs, additional expenditures accrue for travel and medicines. Because of a shortage of drugs for free distribution in public facilities, patients are usually required to purchase prescribed medicines from private pharmacies, which results in higher costs. This reduces the comparative cost advantages of public hospitals.

It was also reported that NDAPs consider the financial situation of their patients, and prescribe cheaper medicines than those dispensed by formally qualified health care providers. In addition, they are said to keep prescriptions flexible, prescribing medicine for two to three days only, and then see whether it works for the patient.

At the same time, many of the discussants think that local providers such as NDAPs are taking advantage of uneducated patients, who do not have any other option than consulting the nearest available practitioner. Though local providers were usually characterized as affordable, some respondents complained about high fees. One person said, “If we consult a local doctor [NDAP], he may call three to four times and make four to five hundred rupees for himself. He charges around hundred rupees at a time. Whenever we call, he prescribes three or four types of medicines. Since we are illiterate, he charges four to five hundred rupees for it” (FGD with male household heads, Vaishali district).

Apart from potentially lower costs of their services, NDAPs offer the possibility of paying in installments and even treat on credit, making their services more affordable. Respondents noted that some NDAPs accept payment in the form of work and advance the fee for treatment at the doctors to whom they refer their patients. Respondents feel that NDAPs offer such payment options to foster good relationships with their clients.

In some instances, patients move from the outside level of the health care system down to local NDAPs. This happens, for example, when formally qualified care becomes too expensive or when patients think that the treatment prescribed by formal providers could be administered more cheaply by NDAPs. As one respondent explains, “Mahua [a bigger town] is nearby, so we go to Mahua, to just get diagnosed. If he [the formally qualified doctor] asks us to take an injection or medicines, in morning and evening, then we go to local doctor and ask him to administer [that] injection” (FGD with male household heads, Vaishali district). Respondents do not always access the best services when seeking health care and “visit that doctor who treat them on credit, whether his medicines suit them or not” (FGD with female SHG members, Kanpur Dehat district).

#### ***Acceptability or cultural access***

The NDAPs’ responsiveness to the financial and service needs of their patients described above are a consequence of their embeddedness in the village communities and an example of their social and cultural accessibility. Some participants of FGDs in the Kanpur Dehat district stressed the importance of relationships between them and their doctor, because one chooses the doctor who has given quick relief in the past and who treats on credit if there is a lack of money. This mutual trust in the ability of the NDAP to provide relief and the honesty of patients to repay their debt is important for the reputation of NDAPs and their success in the community, as respondents said they rely on the recommendations of others when choosing a health care provider. The following quote illustrates how this trust does not always exist when it comes to government hospitals: “Even if the poor [person] is having severe problems, they [at the government hospital] will not pay attention. They will first see these rich people and then call the poor. (…) What I mean to say is, you are a doctor and you should pay attention to the patient irrespective of his caste. (…) We develop faith in a doctor when he is worth of it. You have completed (…) [medical] education, you have a license and what I mean is use it appropriately for poor people. A poor person should not need to tell everything [his symptoms] in a specific manner. You are a doctor and should catch the correct nerve” (FGD with male household heads, Pratapgarh district).

Another aspect of cultural accessibility of NDAPs is how they take into account the belief system of their patients. In many FGDs, respondents confirmed that they consult spiritual healers for their ailments, too, depending on the nature of their disease and the success of alternative treatment. In an FGD with male household heads in the Kanpur Dehat district, it was reported that NDAPs also refer to these spiritual healers when they themselves are unable to cure the disease: “When the medical treatment does not help, then these jholachap doctors [NDAPs] say that this is chakkar [dizziness], show it to some bhagat [a religious devotee]”. As NDAPs are part of the village community themselves, they have adapted the way they provide their services to the villagers’ cultural expectations.

**Figure 2:**
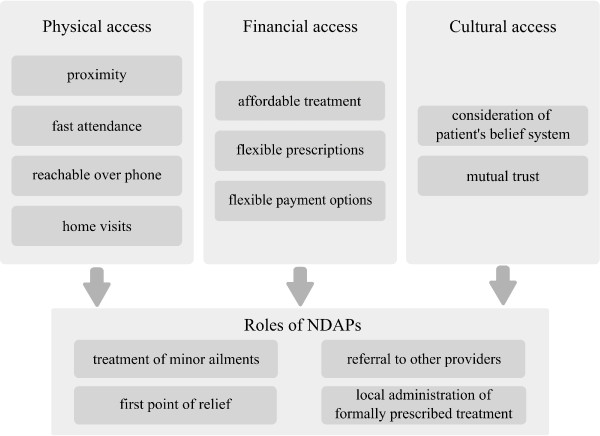
Access to NDAPs and their role in the rural health system.

Figure 2 summarizes our findings on the accessibility of NDAPs and the role they fulfill within the rural health system.

## Discussion

Starting from the ongoing popularity of private non-degree practitioners in rural northern India, we explored the rural poor’s perception and utilization of the rural health care system and the role and accessibility of NDAPs therein. To the best of our knowledge, the present article is the first to do so for northern India since the introduction of the NRHM. Our study reveals that NDAPs are still the most important health care source for primary care in our study sites. This is in keeping with the results of a household survey on the same population, which finds that NDAPs are consulted most often for acute illness episodes [7]. Interestingly, the reasons brought forward for consulting NDAPs are – despite the achievements of NRHM – the same today as Rohde and Viswanathan identified already 30 years ago: they are close by, provide fast and affordable treatment, offer flexible prescription and payment options and take into account patients’ belief system [8]. Embedded in the community, NDAPs have adapted their services to people’s needs, preferences and economic abilities. This leads – in the terminology of Penchansky and Thomas – to a high degree of fit between them and their clients. George and Iyer reported similar findings for NDAPs and other informal health providers in northern Karnataka, India [14].

Our study shows that the community shapes the private supply side of health care through its demand for physically and financially accessible care and its definition of what is culturally acceptable. The typology created by our study population shows that public and private health care by formally qualified providers is not accessible at the local level in rural areas. Health-care-seeking pathways for acute illness episodes through the identified three-level structure do not correspond to the design of the Indian public health sector, as rural patients self-medicate first, then consult unqualified, informal providers and move to formally qualified providers only at a later stage, if at all. The NRHM envisioned trained ASHAs to be the first contact point for any health-related demand, but for the community NDAPs take on this role when acute illness arises. They cure minor ailments and influence their patients’ health-care-seeking behavior by referring or recommending certain providers. Additionally, they offer an interim solution as providers of temporary relief before more formally trained providers can be accessed. We thus confirm Gautham et al.’s findings from southern and eastern Indian states that NDAPs fill a gap in access to care [6]. We go beyond their study when revealing the rural health care system’s three-level structure described above and that NDAPs also locally administer treatment formally prescribed by “outside” doctors. This spares the patient additional expenditures on transport and fees and opportunity costs.

### Limitations of the study

The FGDs were conducted with inhabitants of villages in two districts in Uttar Pradesh and one district in Bihar. Accordingly, our findings cannot simply be generalized to entire districts or states, let alone the whole country. We compared results across study districts and distances to the next PHC/CHC and found that, despite their differences, inhabitants share a common perception and utilization of the rural health care system. This suggests that our results might at least be partially generalizable to larger regions. As mentioned before, our findings from Bihar and Uttar Pradesh also confirm recent insights from southern and eastern Indian states with respect to health-care-seeking pathways, the popularity of NDAPs and their favorable characteristics [6,8,29]. This shows that there are similarities across the borders of the Indian states. We did not consider access to care for chronic illnesses, however. More research is necessary to gain a fuller picture of the role of NDAPs in India generally and for different kinds of illnesses specifically.

Our rural households are privileged compared with the general disadvantaged rural population concerning access to credit, such as for health care, because they can borrow from their SHG on a relatively low interest rate. This might raise doubts regarding the transferability of our findings to comparable economic groups. But these loans are not always available on short notice, which may be necessary in case of acute health emergencies, and thus in all likelihood do not play an important part in access to care for episodes of acute illness. Because of their involvement with local partner organizations that are active in health-related awareness activities, the SHGs might be more aware of preventive care and the necessity of health care by trained providers than the rest of the village population. In choosing which SHGs to interview, we relied on these partner organizations, which might have biased our results in favor of more successful and open groups. Both potential biases would rather lead to an underestimation of problems in accessing health care, however.

Finally, the fact that the local researchers who conducted some of the FGDs are representatives of the local partner organizations might have influenced the SHGs in their ability to discuss some issues. At the same time, their presence might have elicited increased openness from SHG members because of their familiarity with the organization and its staff. As we did not discuss any of the partners’ activities during our FGDs, we assumed that the influence of their staffs’ presence is negligible.

### Potential policy measures

The problem of the low accessibility of public health care by formally qualified providers can be addressed at two levels: 1) tackling the shortcomings of the public sector, and 2) integrating NDAPs into the public health care system.

Most of the shortcomings of the public sector identified in our data were already addressed in the NRHM, but evidently not thoroughly enough. The purchase of medicines prescribed by public doctors continues to be a problem, and an important reason not to consult public providers. Accordingly, it is important that the Indian government works to ensure the supply of public facilities with the necessary medicines and their distribution. Initiatives to improve the availability of quality public providers, so as to make health care by formally qualified providers more “local”, need to be further strengthened as well. Increasing the quality of public health care is another essential measure. But changing prevalent perceptions among the rural poor about the low quality of public care will probably take quite a while.

If the Indian government restricts itself to just these measures, however, it will overlook the complexity of the accessibility issue. Even after the above issues have been properly addressed, NDAPs will have advantages over public health care services: night availability, home visits, flexible prescriptions and payment options and cultural familiarity. ASHAs were envisioned to have some of these characteristics as well, but our study shows that they are consulted mostly for specific conditions. As a large part of health care seeking for acute illness episodes takes place outside the public health care system, governmental measures to improve access to qualified care could go beyond that system and include NDAPs. Demands for new efforts to certify, integrate and regulate NDAPs have been raised since the 1990s, in India and elsewhere [8,12,28,31,32]. Recent approaches in several Indian states to allow degree holders in alternative medical systems (AYUSH) to practice allopathic medicine are first steps in satisfying these demands [44]. But integrative efforts should not be restricted to AYUSH degree holders; they must include all kinds of NDAPs, who already fulfill tasks of first contact points for primary care. Regulations and formal certification procedures could be introduced that permit NDAPs to treat those illnesses they are able to handle, refer those that are beyond their ability to other providers, provide interim primary care until patients have the necessary means to access higher level health care and administer simpler therapies prescribed by trained doctors. One must consider, however, that regulation and certification will change the way NDAPs provide care, such as by reducing their flexibility in treatment and fees. This, in turn, might decrease their popularity in the community. Accordingly, any interventions must be carefully designed so as not to compromise the NDAPs’ appeal for patients and providers. Past experiences in India with integrating NDAPs into the public health system have shown that the new regulations should, for example, continue to permit private practice [8].

## Conclusions

Our rural study population, located in two of the high-focus states of the NRHM in northern India, developed an informal three-level hierarchical structure of the rural health care system, which patients usually follow from the lower to higher levels for acute illness episodes. Respondents perceive themselves as being isolated from quality health care and restricted to providers in their immediate proximity, because though they view outside care as well-qualified, it is often far away, expensive and culturally unfamiliar. Accordingly, they mostly seek care from local NDAPs because these services better fit rural communities’ needs and preferences. Because they are embedded in the community, NDAPs are more physically, financially and culturally accessible for acute illness episodes, despite the achievements of the NRHM. The Indian government could turn these providers into formally trained and regulated entry points to the public rural health system, comparable to what ASHAs were envisioned to be, but with enhanced capacities such as treatment for a larger variety of acute ailments. It should be kept in mind, however, that such measures would affect the way these providers work, possibly decreasing their accessibility for the rural poor. Consultation of the community, appropriate policies and accompanying research should be instituted to monitor possible changes in, and consequences for, health-care-seeking behavior.

## Endnotes

^a^There have been recent efforts in some Indian states to allow AYUSH doctors to practice allopathy as well [44].

^b^Following Peters et al., we understand quality of services as a component of all access-related dimensions [18], and hence do not treat it as a separate entity.

## Abbreviations

ASHA: Accredited Social Health Activist; AYUSH: Ayurveda, Yoga and Naturopathy, Unani, Siddha and Homeopathy; CBHI: Community-Based Health Insurance; CHC: Community Health Center; FGD: Focus Group Discussion; NRHM: National Rural Health Mission; PHC: Primary Health Center; NDAP: Non-degree Allopathic Practitioner; SHG: Self-Help Group.

## Competing interests

The authors declare that they have no competing interests.

## Authors’ contributions

CM analyzed the data and drafted the manuscript. KR participated in initial steps of data analysis. KR and PP helped in drafting the manuscript. PP managed the data collection and provided oversight for study implementation. All authors participated in the design of the study and read and approved the final manuscript.

## Pre-publication history

The pre-publication history for this paper can be accessed here:

http://www.biomedcentral.com/1472-6963/14/182/prepub
